# Evidence for Horizontal and Vertical Transmission of Mtr-Mediated Extracellular Electron Transfer among the *Bacteria*

**DOI:** 10.1128/mbio.02904-21

**Published:** 2022-02-01

**Authors:** Isabel R. Baker, Bridget E. Conley, Jeffrey A. Gralnick, Peter R. Girguis

**Affiliations:** a Department of Organismic and Evolutionary Biology, Harvard University, Cambridge, Massachusetts, USA; b BioTechnology Institute, University of Minnesota, St. Paul, Minnesota, USA; c Department of Plant and Microbial Biology, University of Minnesota, St. Paul, Minnesota, USA; University of Washington

**Keywords:** *Shewanella*, electron transport, evolution, gene transfer, iron oxidizers, iron reduction, lithoautotrophic metabolism, phylogenetic analysis

## Abstract

Some bacteria and archaea have evolved the means to use extracellular electron donors and acceptors for energy metabolism, a phenomenon broadly known as extracellular electron transfer (EET). One such EET mechanism is the transmembrane electron conduit MtrCAB, which has been shown to transfer electrons derived from metabolic substrates to electron acceptors, like Fe(III) and Mn(IV) oxides, outside the cell. Although most studies of MtrCAB-mediated EET have been conducted in Shewanella oneidensis MR-1, recent investigations in *Vibrio* and *Aeromonas* species have revealed that the electron-donating proteins that support MtrCAB in *Shewanella* are not as representative as previously thought. This begs the question of how widespread the capacity for MtrCAB-mediated EET is, the changes it has accrued in different lineages, and where these lineages persist today. Here, we employed a phylogenetic and comparative genomics approach to identify the MtrCAB system across all domains of life. We found *mtrCAB* in the genomes of numerous diverse Bacteria from a wide range of environments, and the patterns therein strongly suggest that *mtrCAB* was distributed through both horizontal and subsequent vertical transmission, and with some cases indicating downstream modular diversification of both its core and accessory components. Our data point to an emerging evolutionary story about metal-oxidizing and -reducing metabolism, demonstrates that this capacity for EET has broad relevance to a diversity of taxa and the biogeochemical cycles they drive, and lays the foundation for further studies to shed light on how this mechanism may have coevolved with Earth’s redox landscape.

## INTRODUCTION

Bacteria and archaea are the biological drivers of Earth’s ecological and geochemical evolution (1). Their far-reaching impact on our planet is rooted in their incredible physiological diversity. They are found in every habitat on Earth, defining the edges of the biosphere. One of their metabolic capabilities is the breadth of substrates they can use to harness energy. Some bacteria and archaea have even evolved the means to use exogenous electron donors and acceptors for energy metabolism, such as reduced and oxidized iron-containing minerals. This phenomenon is broadly known as extracellular electron transfer (EET). EET has been implicated as a major agent of environmental change, including the oxidation of methane (a potent greenhouse gas [2–5]), the rise of oxygen on early Earth (6–9), and the remediation of materials considered toxic to most other forms of life (10–13). EET can occur in both the reductive or oxidative direction depending on the microorganism and source of electrons. However, extracellular redox reactions are accompanied by a unique physiological challenge: electrons must be efficiently transferred between environment and cell across insulating protective barriers.

Several seemingly independently evolved modes of EET have been identified in a range of microorganisms (5, 14–24). Of these, two taxa have become the primary models for studying the biochemistry and physiology of EET: *Shewanella* species EET and *Geobacter* species EET in *Shewanella* spp. (25–27) are mediated by the MtrCAB system, in which electrons derived from metabolic activity are transported from the inner membrane through a tetraheme quinol dehydrogenase, CymA, to periplasmic cytochromes CctA or FccA, which then deliver electrons to the decaheme *c*-type cytochrome MtrA, insulated within the beta-barrel protein MtrB located in the outer membrane (28). The MtrAB complex transmits electrons to the extracellular decaheme cytochrome MtrC, which donates those electrons to an electron acceptor outside the cell (28). Most investigations of MtrCAB have focused on its physiology and biochemistry in Shewanella oneidensis MR-1, which was originally isolated as an iron and manganese reducer but has since been shown to employ MtrCAB when respiring other electron acceptors, such as electrodes, chromium, cobalt, technetium, uranium, and vanadium (29). The genes encoding this metabolic capacity are clustered together in the order of *mtrC*, *mtrA*, and *mtrB* in the S. oneidensis MR-1 genome. Immediately upstream of *mtrCAB* is *omcA* (a homolog of *mtrC*), which is preceded by the genes *mtrD*, *mtrE*, and *mtrF*, homologs of *mtrA*, *mtrB*, and *mtrC*, respectively (30).

Recent genomic analyses identified homologs of MtrCAB in *Aeromonas* and *Vibrio* spp. (27, 31, 32), and subsequent functional experiments confirmed that MtrCAB is essential for metal reduction in examined representatives Aeromonas hydrophila and Vibrio natriegens (33, 34). While the MtrCAB complex is conserved in metal-reducing *Shewanella*, *Aeromonas*, and *Vibrio* spp., the inner membrane quinol dehydrogenase and periplasmic electron carrier proteins differ among these three genera, indicating that the *Shewanella* model of the Mtr pathway is not as canonical as previously thought. In addition to other Gammaproteobacteria, homologs of MtrA and MtrB (deemed PioA and PioB, respectively) in the phototrophic alphaproteobacterium Rhodopseudomonas palustris TIE-1 are required for EET in the opposite direction; that is, electrons travel from outside to inside the cell while oxidizing extracellular donors like iron (Fe^2+^) or cathodes (35–37). Other homologs of MtrAB called MtoAB have also been proposed to function in chemolithoautotrophic iron oxidation by the betaproteobacteria *Gallionella* spp. and *Sideroxydans* spp. (38–41).

These examples of Mtr-linked EET activity found among diverse taxa within the *Bacteria* point to a shared lineage, the evolution of which could be resolved by knowing how widespread this metabolic capacity is throughout the tree of life. In light of the massive increase in the number of microbial genomes and recent advances in computational tools for analyzing patterns across genomes, we posit that a new survey of the available genomic data paired with careful phylogenetic analysis could (i) better determine how widespread Mtr-mediated EET is among contemporary taxa, (ii) reveal the scope of this system’s variations, and (iii) reveal connections between MtrCAB’s evolution, function, and impact on the environment. Such an effort would represent a significant advance, building upon previous studies (25–27, 32, 40, 42–44) that examine the evolution and/or distribution of MtrCAB and related pathways.

Here we employ a phylogenetic and comparative genomics approach to look for Mtr-mediated EET across all three domains of life. We find *mtrCAB* in the genomes of numerous diverse bacteria from a wide range of environments, including among taxa from entire classes and even phyla that, to our knowledge, have never been shown to encode MtrCAB until now. The data further suggest that *mtrCAB* has been transmitted through several horizontal gene transfer (HGT) events, each followed by modular diversification of both its core and accessory components. Our findings point to an emerging story about the evolution of EET and the capacity for extracellular metal-oxidizing and -reducing metabolism and lay the foundation to resolve how this mechanism may have coevolved with Earth’s redox landscape and inform biogeochemical models that implicate EET.

## RESULTS

### The capacity for MtrCAB-mediated EET is widespread among phylogenetically and physiologically diverse Bacteria.

**FIG 1:**
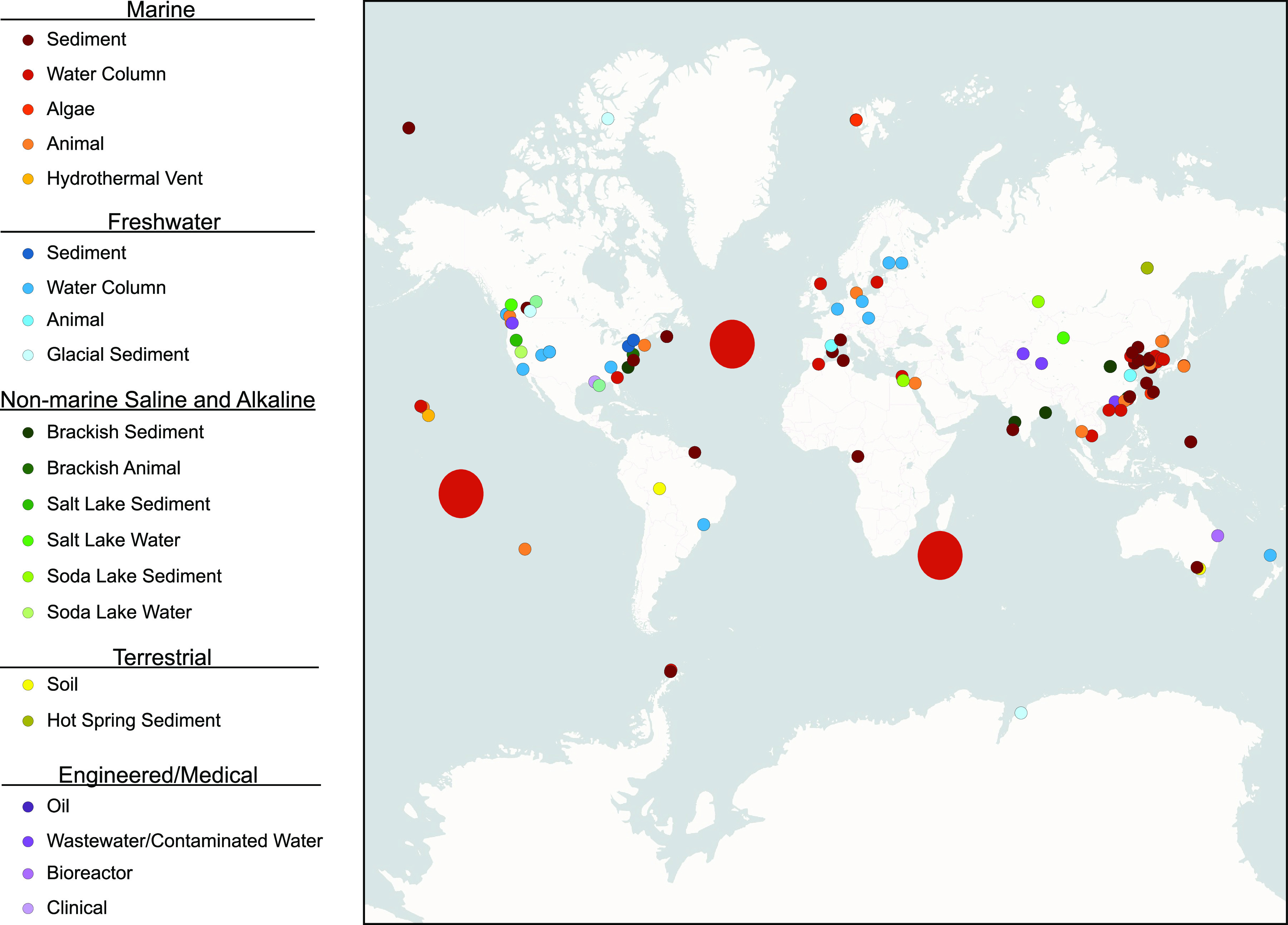
Geographic locales of microorganisms encoding elements of the MtrCAB system. The geographic location of isolation was unavailable for some sequences, and geographical sampling biases are apparent. Large red circles represent the South Pacific, North Atlantic, and Indian Ocean (Eastern Africa Coastal Province) regions described by Tully et al. (146). For more details, see Table S1. The map was created using the Positron base map available in QGIS (https://cartodb.com/basemaps/) (map tiles by CartoDB, under CC BY 3.0. Data by OpenStreetMap, under ODbL).

The MtrCAB outer membrane conduit has been genetically and physiologically implicated in EET among Shewanella oneidensis, *Shewanella* sp. ANA-3, Aeromonas hydrophila, and Vibrio natriegens (28, 31, 33, 34, 45, 46). Accordingly, to begin investigating the prevalence of these genes among other bacteria, we searched for homologs of MtrCAB across the entire domain. Given that the MtrCAB-encoding genes are directly adjacent to each other in the aforementioned models, we constrained our search to include only hits in which *mtrC*, *mtrA*, and *mtrB* occur as a cluster, in any order, in a genome (see Materials and Methods for more details). With these parameters, we found that MtrCAB is encoded in numerous phylogenetically diverse Bacteria, spanning 148 species representing 13 orders, 5 classes, and 3 phyla. Most of the species identified here belong to the Gammaproteobacteria and Betaproteobacteria, in addition to 5 hits among the Acidobacteriia and singletons from the Alphaproteobacteria and Gemmatimonadetes. These classes are composed exclusively of Gram-negative bacteria, with neither eukaryotes nor archaeans predicted to encode MtrCAB. These observations suggest that MtrCAB-mediated EET is restricted to Bacteria with an outer membrane, likely evolving after the divergence of *Archaea* and Bacteria from the last universal common ancestor (LUCA). Parallel with these various environmental contexts and taxonomic affinities, the species predicted to encode MtrCAB include those described as chemoorganotrophs, photoheterotrophs, and chemolithoautotrophs capable of aerobic, facultative anaerobic, and/or fermentative respiratory strategies (see Table S1 in the supplemental material). These 148 MtrCAB-encoding species were recovered from a wide range of environments, including the waters and sediments from both freshwater and marine settings, hot springs and hydrothermal vents, soda and salt lakes, contaminated wastewater, engineered systems, and host-associated habitats (Fig. 1; Table S1) despite the observed geographical sampling biases. Notably, 29 of these species have been directly implicated in some form of EET, especially in the reduction of iron and manganese oxides (Table S1), although it must be noted that MtrCAB was not explored as the explicit driver of EET in most of these cases. While the majority of these cases belong to the *Shewanellaceae* and close relative *Ferrimonadaceae*, they also include Aeromonas hydrophila and Vibrio natriegens (33, 34). Specifically, evidence for EET was also found in a few of the MtrCAB-encoding Betaproteobacteria identified in our search, including the iron reducer Albidoferax ferrireducens T118 (47, 48) (basonym Rhodoferax ferrireducens) and the recently described Ramlibacter lithotrophicus RBP-2, which has been implicated in oxidative EET and expresses *mtrCAB* when grown on Mn(II) in coculture with “*Candidatus* Manganitrophus noduliformans” (49). Other species found to encode MtrCAB, such as *Burkholderiales* bacterium JOSHI_001 and *Ideonella* sp. A288, have been reported to deposit manganese and iron oxides, respectively, but whether or not they yield energy from these reactions remains to be seen (50–52). Likewise, some of the MtrCAB-encoding organisms come from metagenomic samples in which EET was implicated through bioelectrochemical experiments or other geochemical observations, but in lieu of experimental validation for individual genotypes, we chose not to speculate on whether the organisms we identified in these cases are directly engaged in EET.

10.1128/mBio.02904-21.7 TABLE S1 (A) Contextual information for species encoding MtrCAB; (B and C) sequence identifiers for protein and protein-coding sequences used in this study; (D) summary of genetic and biochemical investigations of MtrCAB/DEF; (E) changes made to species names with reasons for modification. Download Table S1, XLSX file, 0.2 MB. Copyright © 2022 Baker et al. 2022 Baker et al. https://creativecommons.org/licenses/by/4.0/ This content is distributed under the terms of the Creative Commons Attribution 4.0 International license.

Beyond these instances where EET has been directly or indirectly implicated, the majority of MtrCAB-encoding organisms have never been experimentally tested for this metabolic capacity. While the MtrCAB system is typically associated with reductive EET (as opposed to oxidative EET like the PioAB system [35, 37]), MtrCAB has been shown to operate as an oxidizing system in artificial lab settings (53, 54). Thus, we cannot infer the net direction of electron flow for most microorganisms identified in our search, because the inherent physiological and environmental controls on electron flow directionality are still poorly understood. Interestingly, one MtrCAB-encoding organism from a metagenome, *Gallionellales* bacterium RIFCSPLOWO2_02_FULL_59_110, is a member of the same suborder as the iron-oxidizing bacteria Gallionella capsiferriformans ES-2 and Sideroxydans lithotrophicus ES-1 that encode homologs of MtrAB but lack MtrC (40, 41).

Moreover, nearly 40% of the species we recovered are members of the *Shewanella*-*Paraferrimonas*-*Ferrimonas* group, for which almost all of the genome assemblies that we analyzed included the MtrCAB gene cluster (Table S2). Vertical transmission of MtrCAB within the *Shewanella-Paraferrimonas-Ferrimonas* group would be consistent with the observed patterns of inheritance, although further phylogenetic work resolving the relationships between these genera is required, as discussed below. Outside of the *Shewanella-Paraferrimonas-Ferrimonas* group, the remaining species in which we did identify *mtrCAB* are not unique to a single bacterial clade; those that do possess MtrCAB are generally the minority among their genus, family, order, or even class or phylum (Table S2). Horizontal gene transfer (HGT) is one mechanism that could explain the sporadic phylogenetic representation among the species encoding MtrCAB; the fact that we find the *mtrCAB* gene cluster scattered among many taxonomically divergent species is inconsistent with vertical transmission as the sole agent driving *mtrCAB*’s distribution.

10.1128/mBio.02904-21.8 TABLE S2 Taxonomic classification for species encoding MtrCAB and the fraction of species in these genera that encode MtrCAB. The number of total available assemblies assessed reflects those assemblies available in the NCBI database on or before 8 July 2020. Only 1 strain per species was included in generating the number of total assemblies available for a genus. This includes species designated “sp.,” even without a strain identifier, although exceptions were made for genera that are disproportionately sequenced at much higher frequencies. These genera were *Aeromonas*, in which species named “*Aeromonas* sp.” were counted only if they had a strain designation, as well as *Shewanella* and *Vibrio*, for which any assemblies without a species designation (i.e., *Shewanella* sp. or *Vibrio* sp.) were not counted, regardless of strain information. Download Table S2, XLSX file, 0.02 MB. Copyright © 2022 Baker et al. 2022 Baker et al. https://creativecommons.org/licenses/by/4.0/ This content is distributed under the terms of the Creative Commons Attribution 4.0 International license.

### Relationships between MtrCAB sequences are incongruent with species phylogeny.

Beyond antibiotic resistance, HGT has been shown to mobilize metabolic pathways, such as genes encoding chlorate reduction (55, 56), perchlorate reduction (57), and photosynthesis (58, 59). We hypothesized that the capacity for MtrCAB-mediated EET was horizontally transferred based on the breadth and scattering of phylogenetic diversity in our curated search. Since incongruent phylogenetic relationships between gene and species trees are a hallmark of HGT (60), we built a tree of the identified MtrCAB sequences to test our hypothesis. In addition to building individual MtrA/D, MtrB/E, and MtrC/F trees, we also concatenated the MtrA(D), MtrB(E), and MtrC(F) sequences for each identified cluster and used these concatenated sequences to build a maximum likelihood (ML) tree.

**FIG 2:**
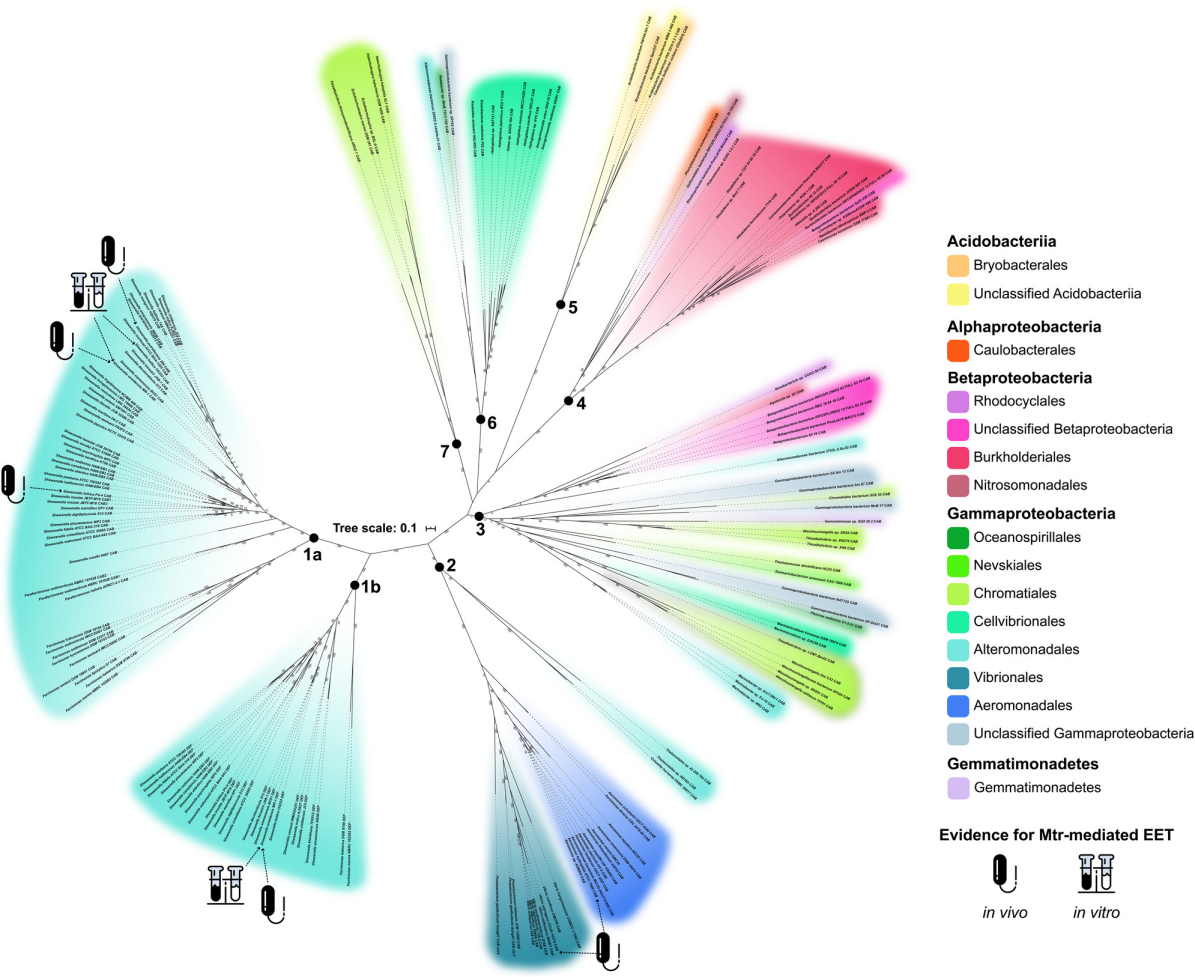
Phylogenomic relationships among MtrCAB coding sequences. This maximum likelihood tree contains 177 concatenated MtrA(D), MtrB(E), and MtrC(F) amino acid sequences encoded in the genomes of 148 species. Each node represents a single concatenated MtrCAB(MtrDEF) sequence. Color codes were assigned by taxonomic order. Bootstrap values are indicated along branch points. Bold numbers 1 to 7 indicate MtrCAB groups referenced throughout this paper. Groups 1a and 1b represent MtrCAB and MtrDEF, respectively, in the *Shewanella* spp. and *Ferrimonadaceae*. Sequences derived from species with previous evidence of MtrCAB/DEF-dependent EET are noted in the “Investigations of Mtr” section in Table S1. Genetic, *in vivo* evidence is denoted with a bacterium symbol, and biochemical, *in vitro* evidence is denoted with a test tube symbol.

The results of the MtrCAB maximum likelihood tree (Fig. 2) imply seven distinct clades, or diversifications, of the MtrCAB system, herein referenced as Groups 1 to 7. Group 1 is composed mostly of *Shewanella* spp., with the remainder representing the closely related family *Ferrimonadaceae* (61, 62). The majority of species represented in Group 1 had both MtrCAB and its paralog MtrDEF, which were incorporated into the tree-building process as separate sequences. MtrCAB and MtrDEF formed separate clades on the tree and are distinguished as Groups 1a and 1b, respectively. Group 2 contains MtrCAB sequences mostly from species of *Vibrio* and *Aeromonas* in addition to a few *Photobacterium*, *Thalassotalea*, and *Colwellia* species. While Group 3 did not include any experimentally validated cases of EET, it was the most phylogenetically diverse cluster, representing numerous *Thioalkalkivibrio* spp. and *Wenzhouxiangella* spp., *Marinobacter* spp., unclassified Gammaproteobacteria and Betaproteobacteria, and a member of the phylum Gemmatimonadetes. Group 4 is almost completely populated by *Betaproteobacteria*, most of which are Burkholderiales, as well as singletons from the unclassified Betaproteobacteria, the orders Rhodocyclales and Nitrosomonadales (Gallionellales), and 1 Alphaproteobacterium from the order *Caulobacterales*. Group 5 is made up exclusively of sequences from the Acidobacteriia that are unclassified or belong to the order Bryobacterales. Sequences from the Cellvibrionales *Halieaceae* family comprised the majority of Group 6, with single additional representatives from Oceanospirillales, Alteromonadales, and unclassified Gammaproteobacteria. Lastly, Group 7 contained MtrCAB sequences from the family *Ectothiorhodospiraceae*.

By and large, the MtrCAB gene tree does not align with the phylogenetic relationships of the species predicted to encode MtrCAB identified here, consistent with the hypothesis that HGT played a role in the dispersal of MtrCAB to the species represented in Fig. 2. These apparent relationships between MtrCAB clades are generally mirrored in the individual MtrA, MtrB, and MtrC trees (Fig. S1 to S3). Examples of phylogenetic incongruences are most apparent in Group 3, which includes distantly related taxa belonging to the Betaproteobacteria, Gammaproteobacteria, and Gemmatimonadetes. While the Betaproteobacteria and Gammaproteobacteria do form distinct clades within Group 3 (excluding MtrCAB from *Alteromonadaceae* bacterium 2753L.S.0a.02 and *Gemmatimonas* sp. SG8_38_2, which groups with the Betaproteobacteria and Gammaproteobacteria, respectively), the relationships among MtrCAB within these classes are largely discordant with their host species’ relationships. Within the Group 3 Gammaproteobacteria, for example, one subset of MtrCAB from *Thioalkalivibrio* and *Wenzhouxiangella* sp. (order Chromatiales) appears to be a sister to MtrCAB from *Marinimicrobium* sp. (order Cellvibrionales), while another subset of the same Chromatiales genera group with *Gemmatimonas* sp. SG8_38_2, which is not a proteobacterium at all but rather a member of the phylum Gemmatimonadetes (63). Another example of MtrCAB species phylogeny incongruence is the Alteromonadales species (*Colwellia and Thalassotalea* spp.) represented in Group 2, which is otherwise comprised of sister orders Vibrionales and Aeromonadales (64, 65). A similar instance can be found in Group 6, which features MtrCAB from Alteromonadales, Oceanospirillales, and an unclassified Gammaproteobacterium in an otherwise *Halieaceae*-dominated group. One possible caveat, however, is that these long phylogenetic distances may be an artifact of a lack of representative extant sequences.

10.1128/mBio.02904-21.1 FIG S1 MtrC maximum likelihood tree generated from amino acid sequences encoded by all core *mtrC(mtrF)* genes (those encoded directly adjacent to *mtrAB*) as well as those *mtrC* genes next to core *mtrCAB* clusters. Numbered, colored circles correspond to numbered, colored arrows in Fig. 6. Bootstrap values are indicated along branch points. Download FIG S1, EPS file, 9.3 MB. Copyright © 2022 Baker et al. 2022 Baker et al. https://creativecommons.org/licenses/by/4.0/ This content is distributed under the terms of the Creative Commons Attribution 4.0 International license.

Conversely, the high species representation of *Shewanella* in Group 1 (40 MtrCAB-positive species out of the 45 *Shewanella* species with genome assemblies available at the time of this study; see Materials and Methods for details) and the close topological alignment with the *Shewanella* species phylogeny (27, 66) suggest that MtrCAB is vertically transmitted among *Shewanella* spp. The *Ferrimonadaceae* represented in Group 1 also suggest a history of *mtrCAB* being vertically transmitted, with all available *Ferrimonas* genome assemblies and 2 out of 3 *Paraferrimonas* genomes encoding MtrCAB. However, the family-level relationships between the *Shewanellaceae* and *Ferrimonadaceae* still require further resolution; some studies point toward the *Shewanellaceae* and *Ferrimonadaceae* being sisters to one another (61, 67), while other studies suggest that the *Shewanellaceae* are in fact more closely related to the MtrCAB-lacking *Moritellaceae* than they are to the *Ferrimonadaceae* (68, 69). Additionally, the *Shewanellaceae* species *Psychrobium* and *Parashewanella* do not encode MtrCAB. Thus, it is not yet possible to resolve the evolutionary order of events that led to the transmission of MtrCAB among the *Shewanellaceae* and *Ferrimonadaceae*; it is possible that the *Shewanellaceae*-*Moritellaceae*-*Ferrimonadaceae* ancestor possessed *mtrCAB* but that it was later lost in the *Moritellaceae* lineage or that separate HGT events led to the *Shewanellaceae* and *Ferrimonadaceae* ancestors acquiring *mtrCAB* separately. This latter scenario does not preclude the possibility that the ancestor of either of these two families transferred *mtrCAB* via HGT to the other.

Beyond phylogenetic discrepancies in the individual MtrCAB groups, the overall diversity and topology of the MtrCAB tree suggest a complicated evolutionary history driven in large part by HGT. With the exception of the *Shewanellaceae* and *Ferrimonadaceae*, the species predicted to encode MtrCAB are not representative of the majority of their sequenced representatives; that is, few or no other members of the same taxonomic group encode MtrCAB. It has been previously noted, for example, that the genetic potential to perform EET is unevenly dispersed within the genera *Aeromonas* and *Vibrio*, with certain clades maintaining *mtrCAB* predominantly in the genome and other strains as the single MtrCAB-encoding representative (33, 34). This sporadic representation of MtrCAB-encoding species suggests two potential evolutionary histories: multiple secondary losses in the majority of lineages or, more likely, insertion into the genomes of the strains we identified in our search.

### Genomic comparisons suggest that *mtrCAB* is highly mobile.

**FIG 3:**
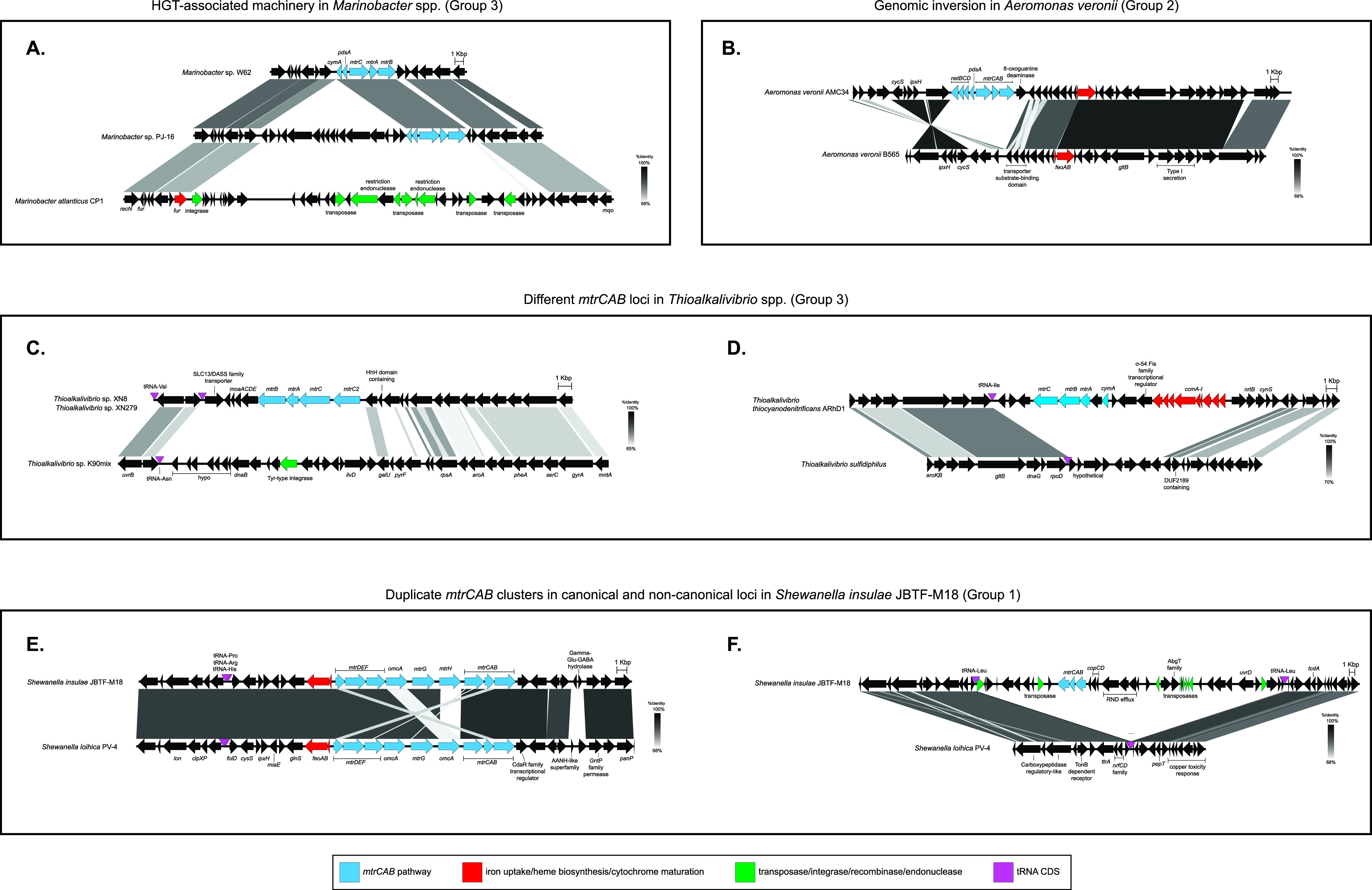
Genomic comparisons of *mtrCAB* loci in MtrCAB-encoding organisms and syntenic regions in MtrCAB-lacking relatives highlights the mobility of *mtrCAB*.

To address the two scenarios mentioned above, we examined the context of the *mtr* locus by comparing genomes of strains carrying *mtrCAB* with genomes from closely related species that apparently lack *mtrCAB*. These comparisons revealed genomic “scars” indicative of events where *mtrCAB* might have been inserted or lost in the past (Fig. 3; Fig. S4). In general, we found that *mtrCAB* seemingly interrupted the otherwise syntenic region in the *mtrCAB*-lacking genome, suggesting that *mtrCAB* was inserted in these sites. In other instances, the aligning regions between two genomes revealed genes encoding transposases, integrases, endonucleases, and/or recombinases in place of *mtrCAB*, perhaps indicating a prior loss of *mtrCAB* from the genome. Below, we describe several representative examples that demonstrate the mobility of *mtrCAB* and linked accessory genes.

10.1128/mBio.02904-21.4 FIG S4 Additional genomic comparisons between *mtrCAB* loci in MtrCAB-encoding organisms and syntenic regions in MtrCAB-lacking relatives. Download FIG S4, EPS file, 1.9 MB. Copyright © 2022 Baker et al. 2022 Baker et al. https://creativecommons.org/licenses/by/4.0/ This content is distributed under the terms of the Creative Commons Attribution 4.0 International license.

One notable example is the *mtrCAB*-lacking genome of Marinobacter atlanticus CP1, a member of a cathodically enriched electroactive community (70–74), which has multiple transposases and restriction endonucleases encoded in the same genomic locus that encodes MtrCAB in fellow *Marinobacter* species, *Marinobacter* sp. W62 and *Marinobacter* sp. PJ-16 (Fig. 3A). In contrast to gene loss evidence based on the presence of mobility-associated elements, alignments of the MtrCAB-encoding Aeromonas veronii AMC34 genome revealed a genomic inversion in the region of an *mtrCAB* insertion relative to the same region in *A. veronii* B565 lacking *mtrCAB* (Fig. 3B). The genomic inversion may indicate a past transposase-mediated event at the *mtrCAB* locus in *A. veronii* AMC34, as genomic inversion can result from recombination between inverted repeated sequences which flank transposable elements (75).

*Thioalkalivibrio* spp. have acquired at least two different homologs of *mtrCAB*. The MtrCAB coding DNA sequence (CDS) from Thioalkalivibrio thiocyanodentrificans ARhD1 lies in a distant clade among other similarly related *Ectothiorhodospiraceae* sequences on the MtrCAB tree (Group 7) (Fig. 2). In contrast, MtrCAB from *Thioalkalivibrio* sp. XN8, XN279, and LCM1.Bin42 fall into a diverse clade (Group 3) (Fig. 2) comprised of *Chromatiaceae* and other gammaproteobacterial sequences. Even within Group 3, MtrCAB from *Thioalkalivibrio* sp. XN8 and XN279 and *Thioalkalivibrio* sp. LCM1.Bin42 branch into discrete clusters with two different sets of *Wenzhouxiangella* spp. In addition to belonging to different groups on the MtrCAB tree, the genomic context of *mtrCAB* is also different between *Thioalkalivibrio* sp. XN8 and XN279, *Thioalkalivibrio* sp. LCM1.Bin42, and *T. thiocyanodentrificans* ARhD1 (Fig. 3C and D). In parallel with these differences, the Mtr genes are arranged as C-A-B in the genomes of *Thioalkalivibrio* sp. XN8, XN279, and LCM1.Bin42 but appear in the order A-B-C in *T. thiocyanodentrificans* ARhD1’s genome. The differences in genomic context and relationship on the MtrCAB tree between these *Ectothiorhodospiraceae* species may indicate two divergent mobile elements targeting different insertion sites within this family of Gammaproteobacteria.

Another example of *mtrCAB* mobility was observed in the genome of *Shewanella insulae* JBTF-M18, which contains two nonsyntenic *mtrCAB* gene clusters. One copy of *mtrCAB* is located in the conserved location upstream of *feoAB*, as observed in the closely related Shewanella loihica PV-4 (Fig. 3E and F). The other *mtrCAB* cluster is flanked by transposase CDS and lacks the additional *mtrC*/*omcA* family homologs that are normally observed in *Shewanella* species *mtrCAB* loci. Additionally, this extraneous cluster is located on a segment of DNA between two tandem tRNA-Leu-encoding genes, which can be recognized by certain transposases as insertion sites (75). That said, both copies of MtrCAB from *S. insulae* share more sequence identity with each other than with any other MtrCAB CDS, suggesting that the mobility of *mtrCAB* in this instance was not from a phylogenetically distant donor but instead may indicate an internal duplication event followed by recombination. A similar phenomenon was observed in the 2 *Photobacterium* species recovered in our search, as both Photobacterium lutimaris JCM 13586 and Photobacterium gaetbulicola Gung47 have *mtrCAB* in the same region on the chromosome, but *P. gaetbulicola* Gung47 has a second copy of *mtrCAB* in a different region on chromosome 2 (Fig. S4).

### (iii) Genomic context reveals passenger genes that mobilize with *mtrCAB*.

**FIG 4:**
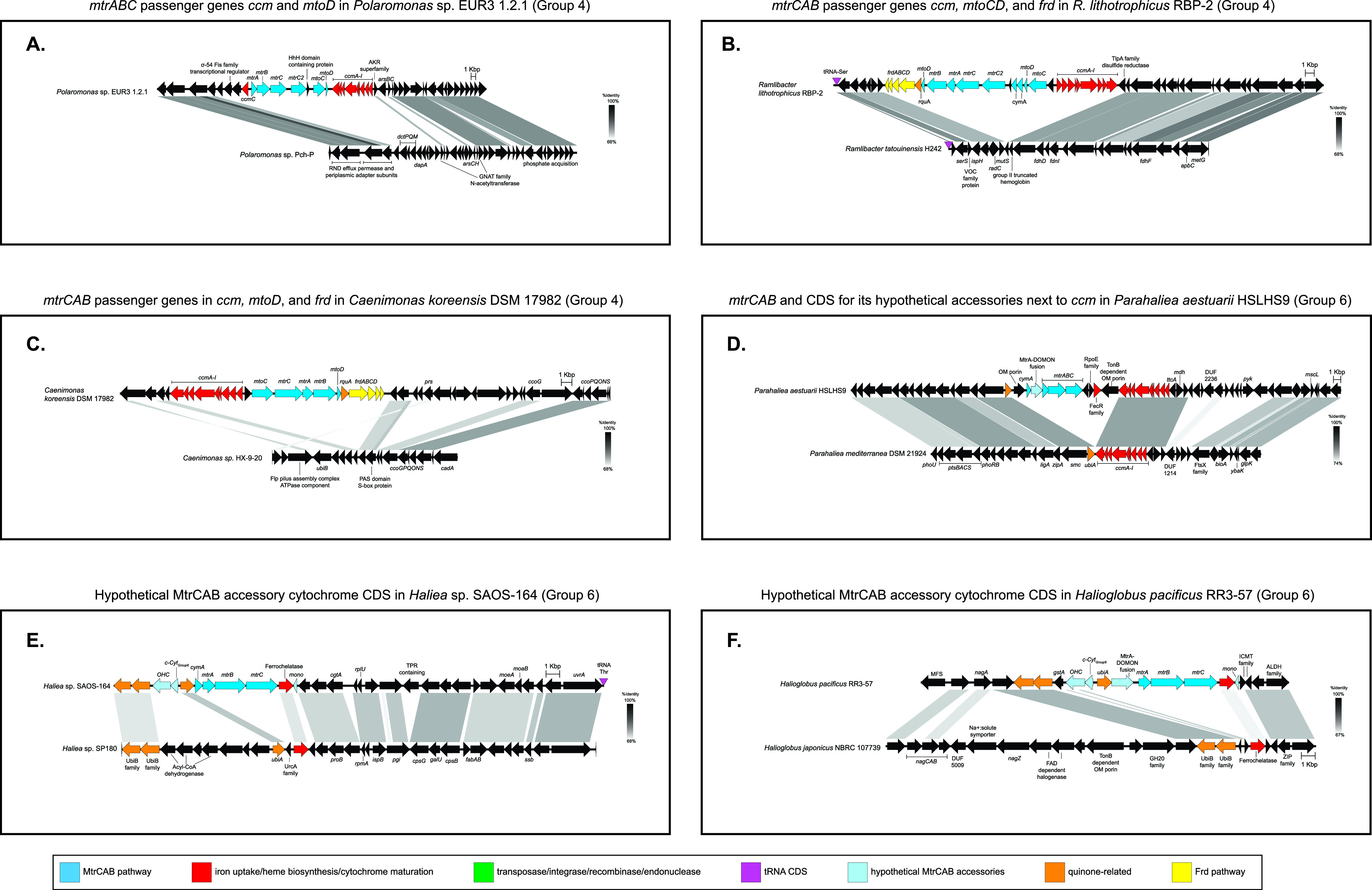
Genomic comparisons of *mtrCAB* loci in MtrCAB-encoding organisms and syntenic regions in MtrCAB-lacking relatives reveal putative *mtrCAB* passenger genes and provide further evidence for *mtrCAB*’s mobility and distribution through HGT.

We noticed that certain genes frequently co-occurred with *mtrCAB* in close genomic proximity, yet these same genes were missing in closely related genomes that lacked *mtrCAB*, suggesting that they may represent auxiliary passenger genes (Fig. 4). The genes therein included other members of the MtrCAB EET pathway that have been functionally characterized (33, 34, 76, 77), such as those coding for the inner membrane quinol dehydrogenases CymA or NetBCD or the periplasmic diheme cytochrome PdsA (Fig. 3A, B, and D; Fig. S4). Transfer of the *mtrCAB/pdsA/cymA*(*netBCD*) gene cluster to another organism would equip the receiving species with a full suite of machinery to perform EET, provided that the receiving genome already contains the appropriate *c*-type cytochrome maturation and menaquinone biosynthetic genes. Other genes clustered with *mtrCAB* included *mtoC* and *mtoD* (38), encoding the putative inner membrane quinone oxidoreductase and periplasmic electron carrier, respectively, that are hypothesized to play a role in extracellular electron uptake in the MtoAB system in Sideroxydans lithotrophicus ES-1 and other related iron-oxidizing Betaproteobacteria (32, 44) (Fig. 4A to C; Fig. S4).

Moreover, we discovered novel putative cytochrome-encoding genes adjacent to *mtrCAB* that are shared among subsets of our newly detected species encoding MtrCAB (Fig. 4D to F; Fig. S4). While the function of these proposed accessory genes is not yet verified, PSORTb (78)-based predictions suggest subcellular localizations for the encoded proteins in the periplasm, outer membrane, inner membrane, or extracellular space, which would be important if they function in the transmission of electrons between the cell and the extracellular environment. Additionally, the fact that these genes are not found in the *mtrCAB*-lacking genomes points toward some level of involvement in the MtrCAB pathway. These features may suggest that certain genes travel together with *mtrCAB*, reminiscent of passenger genes carried by mobile elements (79).

**FIG 5:**
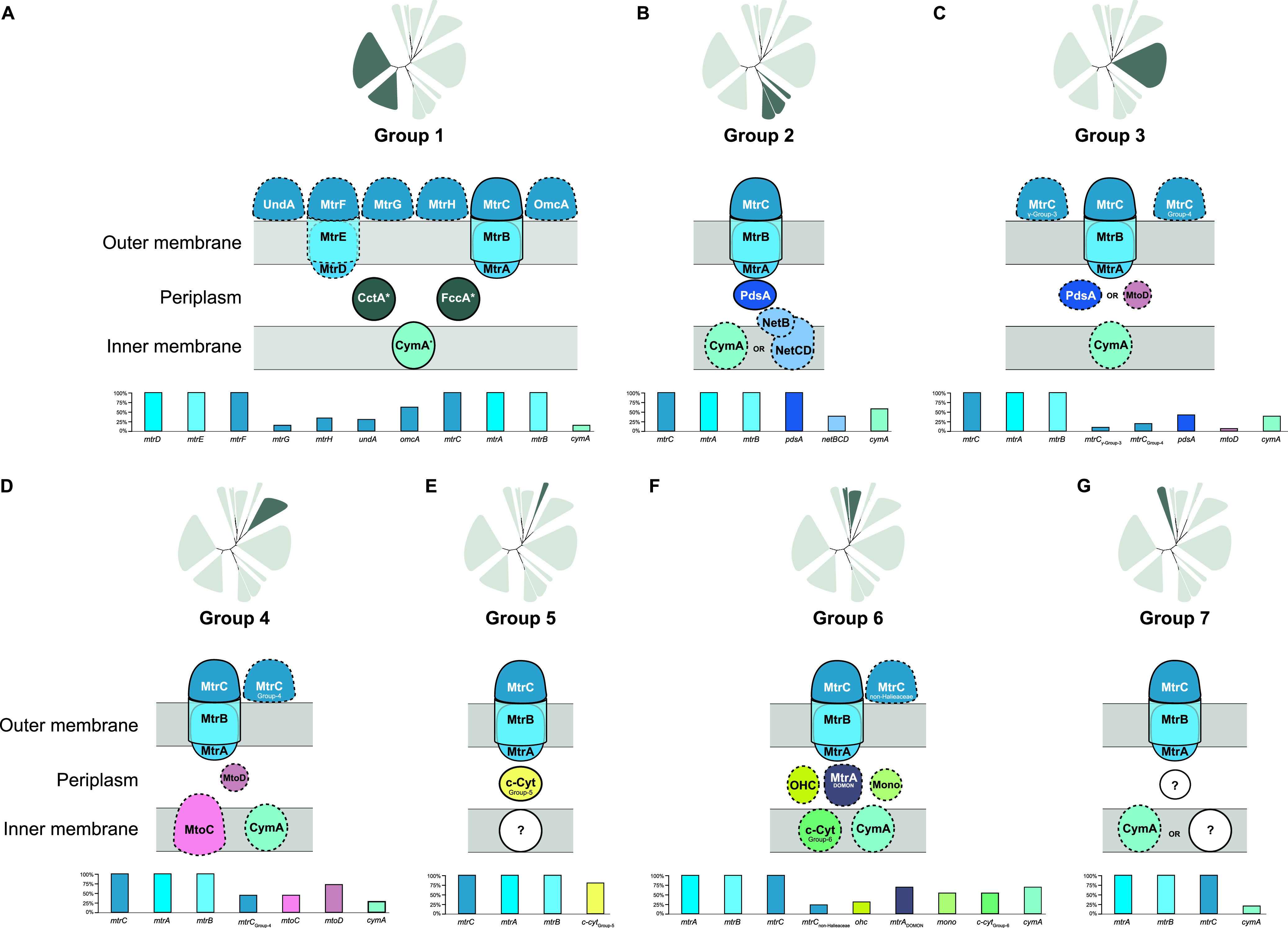
Hypothetical models of MtrCAB and accessory components encoded in *mtrCAB* gene clusters show group-specific diversifications. Protein localization along the cell envelope was predicted with PSORTb (78). Proteins outlined with a solid line are found in every species in the MtrCAB group, while dotted lines indicate that the protein is encoded in at least one but not all members of the MtrCAB group. White circles with a question mark indicate that a putative protein in that cellular location was not encoded in the *mtrCAB* cluster in all or most members of that MtrCAB group. Bar plots show the percentage of members in a given group that encode each MtrCAB component. CymA*, FccA*, and CctA* are not encoded adjacent to the *mtrCAB* cluster in *Shewanella* species nor most *Ferrimonadaceae* species but are included here due to their well-established role in MtrCAB-mediated EET in members of these species. We did not search the genome beyond the identified *mtrCAB* loci for *cymA* or the other depicted accessory cytochromes in other organisms.

To that same end, both verified and putative accessory cytochromes alike—as well as MtrC homologs (see the next section)—seem to align more with MtrCAB tree groups than with organismal phylogeny (Fig. 5). Excluding the Group 1 representatives, which encode CymA and other relevant cytochromes (FccA, CctA) in regions nonsyntenic with the core *mtrCAB* locus, we found that specific cytochromes encoded next to *mtrCAB* were unique to one or two groups, possibly indicating episodic evolutionary events in a group ancestor, suggesting that the MtrCAB evolves in a modular fashion. For example, *pdsA* is found in all members of Group 2 and most Gammaproteobacteria in Group 3 but is absent from other MtrCAB groups (Fig. 3A and B and 5B). Likewise, *mtoC* and *mtoD* homologs are found almost exclusively in the Betaproteobacteria-dominated Group 4 (Fig. 4A to C and 5D), with just one other representative (also a betaproteobacterium) in Group 3 (Fig. 5C) also possessing *mtoD* adjacent to *mtrCAB*. There were also instances of group-specific putative cytochromes that were not found in members of other groups. The Group 5 Acidobacteriia all encoded a predicted periplasmic tetraheme cytochrome (*c-cyt*_group 5_) immediately downstream of *mtrB* (Fig. 5E). Group 6 *mtrCAB* clusters were neighbored by up to 4 encoded cytochromes unique to these *mtrCAB*-encoding species, which to our knowledge have never been described before (Fig. 4D to F and 5F). These include a predicted periplasmic nonaheme MtrA-family cytochrome with a DOMON domain (*mtrA-DOMON*), an inner membrane tetraheme cytochrome (*c-cyt*_group 6_), a periplasmic monoheme protein (*mono*), and a periplasmic octaheme c-type cytochrome (*ohc*). The one commonality to almost all groups was that at least one group member encoded a CymA homolog as part of the *mtrCAB* gene cluster, excluding the Group 5 *Acidobacteriia* which did not encode any putative inner membrane quinone oxidoreductases near the MtrCAB CDS (Fig. 5E).

Mobility of additional EET-associated genes was also observed in multiple *Shewanella* genomes (Fig. S4). In Shewanella sediminis HAW-EB3, for example, CymA is encoded in a conserved location upstream of *menECHD*, as observed in the close relative Shewanella woodyi ATCC 51908 and other MtrCAB-encoding *Shewanella* spp. (Fig. S4). In a separate region of the *S. sediminis* HAW-EB3 genome, however, an additional *cymA* was observed directly next to the periplasmic electron shuttle *pdsA*, the role of which is normally fulfilled by FccA or CctA (80, 81) in the *Shewanella* Mtr pathway (33, 34). Furthermore, the *cymA-pdsA* region in *S. sediminis* (Fig. S4) is flanked by encoded transposases, and both of these *S. sediminis* genes align most closely with homologs from *Vibrio* spp. These genomic features further support the hypothesis of this system being mobile and prone to horizontal transfer, potentially in a modular fashion.

While the majority of sequenced *Shewanella* spp. encode MtrCAB, there are 2 species, Shewanella violacea DSSS12 and Shewanella denitrificans OS217, that do not encode Mtr homologs (Fig. S5) and are unable to reduce extracellular acceptors (82, 83). Given that the other genes involved in or required for the MtrCAB pathway have been especially well studied in *Shewanella* spp., we were able to compare these 2 genomes to their MtrCAB-encoding counterparts to look for further indications of gene loss. A genomic inversion is observed at the site of *cctA* loss in S. denitrificans OS217 (not shown). Loss of *menECHD*, which encodes proteins required for synthesis of menaquinone, was observed in S. denitrificans but not in *S. violacea*, while *cymA* was missing from both species (Fig. S5).

10.1128/mBio.02904-21.5 FIG S5 *mtrCAB* and *cymA* loss in canonical genomic regions in two species of *Shewanella*, a genus in which *mtrCAB* was most likely vertically transmitted. Download FIG S5, EPS file, 1.5 MB. Copyright © 2022 Baker et al. 2022 Baker et al. https://creativecommons.org/licenses/by/4.0/ This content is distributed under the terms of the Creative Commons Attribution 4.0 International license.

Lastly, in addition to electron-carrying cytochromes, alignments between genomes encoding and lacking MtrCAB revealed other potential passenger genes specifically involved in or related to cytochrome synthesis. After all, even if a species encodes MtrCAB, it cannot be utilized without the proper machinery to manufacture and localize functional components of the electron conduit. System I cytochrome maturation, CcmA-I, for example, is essential for maturation of MtrA and MtrC and subsequent EET activity in S. oneidensis (84, 85). System I may have other biological roles besides heme maturation (86) and can serve as a heme reservoir when iron is unavailable for heme synthesis (87). Additionally, system I requires reduction of the oxidized Fe-heme before cytochrome maturation, unlike system II, which protects reduced Fe-heme from oxidation (88) and functions at a lower concentration of iron than system II (89).

Six of the *mtrCAB* gene clusters (Thioalkalivibrio thiocyanodentrificans ARhD1, *Ramlibacter lithotrophicus* RBP-2, *Betaproteobacteria* bacterium SpSt-328, *Polaromonas* sp. EUR3 1.2.1, Parahaliea aestuarii HSLHS9, and Caenimonas koreensis DSM 17982) were genomically adjacent to the complete *ccmA-I* operon (representatives illustrated in Fig. 3D and 4A to D; see also Fig. S4). *Aquincola* sp. S2 and *Thioalkalivibrio* sp. LCM1.Bin42 *mtrCAB* were also neighbored by partial *ccm* operons that were interrupted at the end of a contig. We did not find duplicate system I cytochrome maturation genes in the other genome assemblies encoding CcmA-I next to MtrCAB, although 4 strains (Thioalkalivibrio thiocyanodentrificans ARhD1, *Ramlibacter lithotrophicus* RBP-2, *Betaproteobacteria* bacterium SpSt-328, and *Aquincola* sp. S2) encoded system II cytochrome maturation genes (*ccsAB*/*resBC*) elsewhere on the genome, adjacent to other putative cytochrome-encoding genes. While the rest of these cases indicate that *ccmA-I* is linked with *mtrCAB* in its mobility, this is not the case for *ccmA-I* of Parahaliea aestuarii HSLHS9 (Fig. 4D), which appears to be native to the syntenic region in the *mtrCAB*-lacking Parahaliea mediterranea DSM 21924.

Most of those *mtrCAB* clusters flanked by *ccmA-I* were found in Betaproteobacteria belonging to Group 4. Interestingly, Group 4 *mtrCAB* clusters were also often neighbored by the fumarate reductase complex (*frdABCD*), sometimes in tandem with *ccmA-I* (Fig. 4B and C; Fig. S4). We could not find additional copies of *frdABCD* elsewhere on the genome in these cases, and comparative analyses revealed that *frdABCD* was absent in related genomes lacking *mtrCAB*. Upstream of almost all Group 1 *mtr* clusters is *glnS*, which plays an established role in heme biosynthesis by providing glutamate for the synthesis of the tetrapyrrole precursor 5-aminolevulinic acid (90). The *frd* operon could potentially play a parallel role by providing a source of succinate, which, if converted to succinyl coenzyme A (succinyl-CoA), can also generate 5-aminolevulinic acid (91, 92). Another possible function of FrdABCD in conjunction with Mtr would be to support EET in both the oxidative and reductive directions, an intriguing possibility that warrants further study.

Other genes encoding proteins for heme synthesis, cytochrome maturation, and iron uptake were also observed alongside *mtrCAB* but do not always follow group-specific patterns or are also found in the same region in their *mtrCAB*-lacking relatives, such as with the *ccmA-I* example in Parahaliea aestuarii HSLHS9 detailed above. The menaquinone-dependent protoporphyrinogen IX dehydrogenase gene *hemG* is in the vicinity of *mtrCAB* in *Albidoferax ferrireducens* T118, *Rhodoferax* sp. Bin2_7, and *Betaproteobacteria* bacterium SpSt 328. Gammaproteobacteria bacterium MnB_17 encodes another member of the *hem* operon, oxygen-independent coproporphyrinogen-III oxidase HemN, upstream of its *mtr* gene cluster. The *Marinobacter* sp. Arc7 *mtrCAB* cluster is neighbored by a larger suite of heme synthesis genes (*hemY*, *cysG*, *hemD*, and *hemC*), although these genes are also present in the syntenic region of its *mtrCAB-*lacking relative *M. atlanticus* CP-1 mentioned earlier (Fig. S4). Similarly, all of the Group 6 *mtrCAB* clusters from *Halieaceae* species (excluding Parahaliea aestuarii HSLHS9 and *Halioglobus* sp. NAT121) are immediately upstream of ferrochelatase-encoding *hemH*, which catalyzes the final step of heme synthesis (90, 93), but the same regions identified through alignments of *Halieaceae* species missing *mtrCAB* contain *hemH* as well (Fig. 4E and F).

### Duplications of *mtrC* reveal previous gene flow between *mtrCAB* groups.

In addition to the core *mtrA*, *mtrB*, and *mtrC* genes in each cluster, many *mtrCAB* clusters were neighbored by additional MtrC coding sequences. While only the *mtrC* sequences directly adjacent to *mtrAB* were incorporated into the concatenated MtrCAB tree (Fig. 2), all identified duplicates of *mtrC* proximal to the *mtrCAB* gene cluster were included in building the MtrC tree (Fig. S1). The distribution of these MtrC sequences in different species and on the MtrC tree (Fig. S1) yielded further insights into the transfer and modular evolution of the MtrCAB system.

Of the *mtrCAB* gene clusters identified in this study, 44% had at least one additional *mtrC* immediately next to the core *mtrCAB*. The number of adjacent *mtrC* duplicates clustered with *mtrCAB* ranged from one to as many as four outside of the core *mtrC* directly adjacent to *mtrAB*. That said, *mtrC* duplications were not observed in any members of Group 2 (gammaproteobacteria belonging to *Aeromonadaceae*, *Vibrionaceae*, and *Alteromonadaceae*), Group 5 (*Bryobacteraceae* and other unclassified *Acidobacteriia*), or group 7 (*Ectothiorhodospiraceae* of the *Gammaproteobacteria*). In contrast, every representative of Group 1 (*Shewanellaceae* and *Ferrimonadaceae*) had at least one additional *mtrC* outside of *mtrCAB* and *mtrDEF*, save for 1 of the 2 *mtrCAB* clusters identified in *Shewanella insulae* JBTF-M18 (which likely arose from an internal whole-*mtrCAB* duplication and recombination, discussed in the previous section) as well as the *mtrCAB* from *Shewanella polaris* SM1901. Likewise, the 3 non-*Halieaceae* species in Group 6 have two copies of *mtrC* neighboring *mtrAB*, and 29% and 44% of Groups 3 and 4, respectively, also had representatives with at least one additional *mtrC* family protein neighboring *mtrCAB* in the genome. In many of these instances of duplicate *mtrC* genes, vestiges of prior HGT and diversification events were again revealed by discrepancies between protein phylogeny and taxonomy.

**FIG 6:**
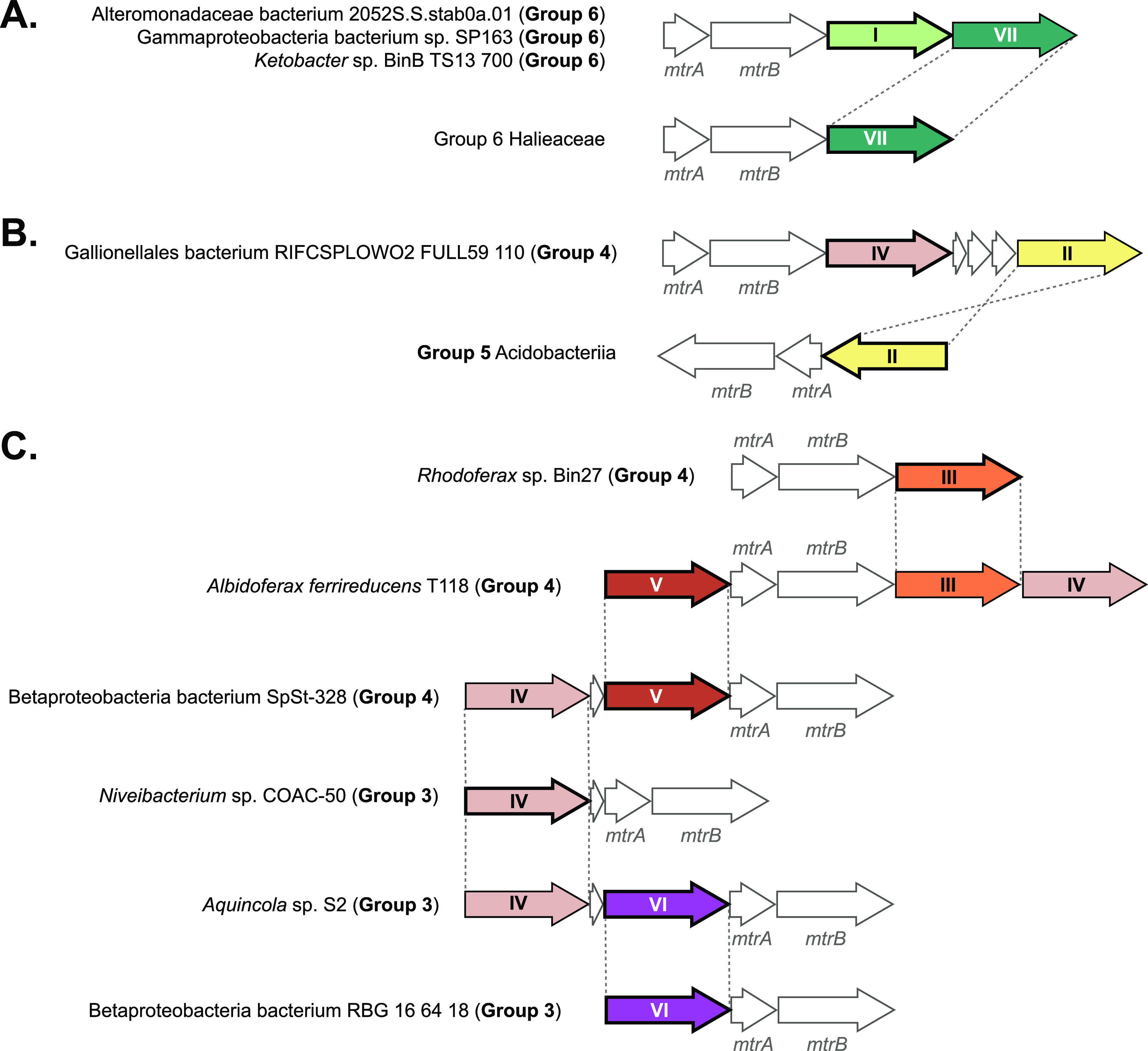
Tracking *mtrC* homologs reveals finer-scale gene flow between MtrCAB-encoding species. Arrows filled with color represent *mtrC* sequences. Colors correspond to the numbered circles (I to VII) in Fig. S1. Arrows with bold outlines indicate the core *mtrC* whose translated coding sequence was incorporated into the concatenated MtrCAB tree (Fig. 2). Unlabeled white arrows in panel C represent a conserved HhH encoded in some *mtrCAB* clusters.

For example, there is a distinct “Group 6” MtrC homolog (designated “VII” in Fig. 6 and Fig. S1) that is present in all Group 6 *mtr* clusters; in the Group 6 *Halieaceae mtr* clusters, this is the sole copy of *mtrC*, and it is immediately downstream of *mtrAB*. In the non-*Halieaceae mtr* clusters in Group 6, however, there is an additional, phylogenetically distinct *mtrC* (designated “non-*Halieaceae* MtrC” in Fig. 5 and “I” in Fig. 6A and Table S1) adjoining *mtrAB* and the Group 6 *mtrC*. The encoded non-*Halieaceae* MtrC appears to be more closely related to the Group 4 and 5 MtrCs (Fig. S1) than to the Group 6 MtrC that is a genomic neighbor. One possible scenario that led to this topology would be the transfer of the Group 6 non-*Halieaceae mtrCAB* cluster to the ancestor of the Group 6 *Halieaceae*, followed by gene loss of the non-*Halieaceae mtrC*. Alternatively, the non-*Halieaceae mtrC* could have arisen as a duplication of the *Halieaceae mtrC* following horizontal transmission from the *Halieaceae* to the non-*Halieaceae* Group 6 members.

Similarly, *Gallionellales* bacterium RIFCSPLOWO2_02_FULL_59_110 (Group 4) encodes 2 copies of MtrC as part of its *mtr* gene cluster. One MtrC (IV in Fig. 6B and Fig. S1) clustered with the corresponding Group 4 Betaproteobacteria MtrC sequences, while the other MtrC (II in Fig. 6B) clustered with Group 5 MtrCs (Fig. S1). These observations could indicate gene flow between Groups 4 and 5 and, based on the lack of other MtrC duplicates in Acidobacteriia, could represent a previous loss event that removed what might have been a functionally irrelevant paralog. Such instances of genome reduction by paralog loss are not uncommon and have been well studied in other systems (94–96) in which a gene is duplicated and subsequently neofunctionalized, followed by a loss of one of the two copies.

There is also clear gene flow and downstream MtrC diversification that connects the Betaproteobacteria of Group 3 with Group 4 (Fig. 5C and 6C; Fig. S1). With the exception of the *Rhodocyclaceae* member *Niveibacterium* sp. COAC-50, the core Mtr found in the Group 3 Betaproteobacteria (VI in Fig. 6C and Fig. S1) groups with another Group 3 MtrC (IV in Fig. 6C and Fig. S1). This is also consistent with the individual MtrA and MtrB trees (Fig. S2 and S3), which place these select Betaproteobacteria among Group 3 (including *Niveibacterium* sp. COAC-50). Interestingly, for *Niveibacterium* sp. COAC-50, the core and sole MtrC (and thus the one incorporated in the concatenated MtrCAB tree) (Fig. 2) clusters with the rest of the Group 4 MtrCs, but the strong affinity of the MtrA and MtrB sequences for Group 3 (Fig. 2; Fig. S2 and Fig. S3) were apparently sufficient enough to overwhelm any Group 4 affinity lent by its MtrC (IV in Fig. 6C and Fig. S1). Group 3 betaproteobacterium *Aquincola* sp. S2 encodes the same Group 4-leaning MtrC (IV in Fig. 6C and Fig. S1) in addition to an MtrC homolog that clusters with other group 3 MtrCs (VI in Fig. 6C and Fig. S1). In both *Niveibacterium* and *Aquincola*, the Group 4-type MtrC coding sequence (IV in Fig. 6C) is followed by a putative gene encoding a helix-hairpin-helix (HhH); in the *Aquincola* genome, this HhH CDS sits between the two *mtrC*s, while it adjoins the sole *mtrC* and *mtrAB* coding sequences in *Niveibacterium*. The retention of this HhH gene beside the Group 4-type *mtrC* and the Group 3-leaning nature of *Niveibacterium*’s *mtrAB* are consistent with a deletion of the Group 3-type *mtrC* in *Niveibacterium*.

10.1128/mBio.02904-21.2 FIG S2 MtrA maximum likelihood tree generated from amino acid sequences encoded by all *mtrA*(*mtrD*) sequences in the *mtrCAB* clusters identified in our study. Homologs of MtrA from chemolithotrophic (MtoA) and phototrophic (PioA) iron-oxidizing bacteria (FeOB) were also included in building the tree and are indicated in red. Bootstrap values are indicated along branch points. Download FIG S2, EPS file, 6.7 MB. Copyright © 2022 Baker et al. 2022 Baker et al. https://creativecommons.org/licenses/by/4.0/ This content is distributed under the terms of the Creative Commons Attribution 4.0 International license.

10.1128/mBio.02904-21.3 FIG S3 MtrB maximum likelihood tree generated from amino acid sequences encoded by all *mtrB*(*mtrE*) sequences in the *mtrCAB* clusters identified in our study. FeOB homologs of MtrB from chemolithotrophic iron-oxidizing betaproteobacteria (labeled MtoB) and a phototrophic iron-oxidizing alphaproteobacterium (labeled PioB) were also included in building the tree and are indicated in red. Bootstrap values are indicated along branch points. Download FIG S3, EPS file, 6.2 MB. Copyright © 2022 Baker et al. 2022 Baker et al. https://creativecommons.org/licenses/by/4.0/ This content is distributed under the terms of the Creative Commons Attribution 4.0 International license.

This apparent deletion, however, is not the only instance of overlap between the Betaproteobacteria of Groups 3 and 4. In fact, many of the Group 3 betaproteobacterial genomes also contain MtrC coding sequences that are not present in any other Group 3 genomes yet are abundant in Group 4. It must be noted, however, that there is one clade of MtrCs each in the Group 3 betaproteobacteria and in Group 4 that are unique to the representatives of the respective *mtrCAB* group genomes. All of the Group 3 Betaproteobacteria (except *Niveibacterium* sp. COAC-50) have a core *mtrC* (VI in Fig. 6C) that is not present in any of the Group 4 genomes. Likewise, a subset of the Group 4 Betaproteobacteria have a core *mtrC* (V in Fig. 6C) that is unique to the Group 3 genomes.

### MtrC has diversified and formed distinct clades in Group 1.

Relative to these other MtrC groupings, the MtrC family proteins encoded in the Group 1 (*Shewanella* spp., *Ferrimonas* spp., and *Paraferrimonas* spp.) *mtrCAB/DEF* gene clusters formed exceptionally distinct clades (Fig. S1 and S6). We named these MtrC family clades (MtrC, MtrF, OmcA, UndA, MtrG, MtrH) based on previously published descriptions and characterizations (80, 97–107); however, there are naming discrepancies in the literature for MtrH, OmcA, and UndA (25, 27, 32, 108). To reconcile these discrepancies, we propose updating the naming conventions for this family of proteins based on our MtrC tree (Fig. S1 and S6; Table S1), which was built from significantly more sequence data than what previous analyses had available at their time of publication (25, 27).

10.1128/mBio.02904-21.6 FIG S6 Genomic arrangement of *mtrC* homologs in group 1 *mtrCAB* clusters. Gene names are based on corresponding placement in the MtrC tree (Fig. S1). Download FIG S6, EPS file, 1.4 MB. Copyright © 2022 Baker et al. 2022 Baker et al. https://creativecommons.org/licenses/by/4.0/ This content is distributed under the terms of the Creative Commons Attribution 4.0 International license.

The number (0 to 5) and subfamily (MtrC, MtrF, OmcA, UndA, MtrG, MtrH) of MtrC family proteins encoded in *mtr* gene clusters varied widely across Group 1, but all species encoded MtrC. MtrC associates with MtrAB at a 1:1 ratio in the outer membrane and is reduced by MtrA (28, 109). Based on structural and sequence homology to MtrC, MtrF likely associates with MtrDE, in a manner similar to MtrCAB (99). MtrF was present in 49% of Group 1 species and was always encoded immediately downstream from *mtrDE*, in the same way that *mtrC* is always observed immediately upstream of *mtrAB* (Fig. S6). MtrF formed a sister clade to MtrC (Fig. S1), recapitulating the relationship observed in the concatenated MtrCAB tree (Fig. 2) and supporting previous hypotheses about their heritage (31). The shared ancestry of both the individual MtrC/MtrF coding sequences and the MtrCAB/MtrDEF clusters indicates that *mtrDEF* and *mtrCAB* formed through a duplication of the entire gene cluster rather than through duplications of the individual *mtrA/D*, *mtrB/E*, and *mtrC/F* genes. However, the order of operations that led to the birth of these two gene clusters—that is, whether *mtrDEF* arose as a duplication and reconfiguration of *mtrCAB* or vice versa—remains to be determined.

Conversely, the other MtrC family proteins that we uncovered in Group 1 (OmcA, UndA, MtrG, MtrH) do not appear to have coevolved with a complementing *mtrAB/DE* but, based on their observed relationships (Fig. S1), have emerged through individual duplications and diversifications of an ancestral MtrC family protein. OmcA was the most common ancillary MtrC family protein encoded in Group 1, with 64% of species carrying at least one copy of *omcA* and several species carrying two nonsyntenic *omcA* homologs (Fig. S1 and S6; Table S1). UndA, another previously reported MtrC family protein (25, 102, 108), was encoded in 30% of Group 1 species and also displayed duplications within some *mtr* gene clusters. *mtrG* and *mtrH* encode uncharacterized MtrC family proteins that are predicted to localize to the extracellular space, like OmcA and UndA, and occurred in 11% and 34% of Group 1 species, respectively.

None of the *mtr* clusters in the Group 1 species encoded all 6 of the MtrC family proteins that we identified. That said, except for *S. polaris* and cluster 2 in *S. insulae* JBTF-M18 (as mentioned at the beginning of this section), all Group 1 clusters included at least one ancillary MtrC family protein. Gene clusters that included both *mtrCAB* and *mtrDEF* always had at least one ancillary MtrC family protein (OmcA, UndA, MtrG, MtrH) encoded between the 2 complete modules. OmcA and UndA were the only MtrC family proteins encoded between *mtrCAB* and *mtrDEF* when only one ancillary MtrC family gene was present. In clusters lacking *mtrDEF*, the MtrC family protein encoded next to *mtrCAB* was either MtrH, OmcA, or UndA but never MtrG. Beyond these parameters, there were no “rules” as to the combination of genes encoding different MtrC family proteins in a given *mtr* cluster (i.e., the presence of a specific clade of MtrC family gene was not dependent on the presence of another).

## DISCUSSION

In this study, we set out to determine the prevalence of *mtrCAB* genes throughout all three domains of the tree of life, with the broader goals of (i) capturing the prevalence of these transmembrane systems among all taxa, (ii) understanding the evolution and mobility of *mtrCAB*-mediated EET, and (iii) providing a roadmap for the empirical assessment of EET among those taxa with the *mtrCAB* genes. With the only requirement being that the *mtrC*, *mtrA*, and *mtrB* genes occur in close succession together in a given genome, we found that the genomic potential for EET is broadly distributed among Gram-negative Bacteria from a wide range of environments and geographic locales (Fig. 1; see Table S1 in the supplemental material). The sporadic phylogenetic representation (Table S2) among various orders of Gammaproteobacteria, Betaproteobacteria, Alphaproteobacteria, Acidobacteriia, and Gemmatimonadetes led us to hypothesize that this system was dispersed largely through horizontal gene transfer. The incongruences in the concatenated MtrCAB phylogenetic tree (Fig. 2) support this hypothesis, as seen in the topology both within and between tree groups. Part of this mismatch between species phylogeny and relationships among MtrCAB coding sequences can be attributed to the fact that the overwhelming majority of genera (excluding *Shewanella* spp. and the *Ferrimonadaceae* in which *mtrCAB* was likely vertically transmitted to all species following an ancestral HGT) represented in our tree contain mostly MtrCAB-lacking species (Table S2), not to mention the genera and orders interspersed between those in our tree in which MtrCAB is completely absent.

That said, these closely related genomes lacking *mtrCAB* afforded us the opportunity to further assess the HGT hypothesis through a comparative genomics approach. These comparisons further supported HGT as the main mechanism by which *mtrCAB* spread. In addition to revealing footprints from prior recombination or transposition events (Fig. 3 and 4), this method revealed putative genes that are linked with MtrCAB, potentially as passenger genes, should *mtrCAB* comprise a mobile element as our data suggest. In addition to genes likely associated with maturation of MtrCAB and its associates, analysis of the genes neighboring *mtrCAB* revealed coding sequences for putative hemoproteins predicted to localize along the cell envelope (Fig. 3 and 5). These include periplasmic and inner membrane electron carriers with established functions in some species (i.e., CymA, PdsA, NetBCD), as well as proteins implicated in iron oxidation (i.e., MtoC, MtoD), and other putative cytochromes that, to our knowledge, have not been reported before. The fact that some of these hypothesized ancillary MtrCAB components are group-specific (Fig. 5) strongly suggests that these components coevolved with MtrCAB and highlight the capacity for MtrCAB and its accessories to change in a modular fashion. This also includes the duplications and diversification of MtrC (Fig. 6).

In the following sections, we address two pressing questions that were prompted by our findings: (i) what evolutionary events in the past led to the relationships among MtrCAB modules observed today, and (ii) do the modular innovations associated with MtrCAB reflect adaptations to the environments in which they emerged?

### An emerging evolutionary story.

The relationships between MtrCAB coding sequences (Fig. 2; Fig. S1 to S3), largely confounded by their incongruencies with species phylogeny, do not lend themselves to an especially clear portrait of their evolutionary history. As discussed throughout this paper, we hypothesize that *mtrCAB* comprises a mobile genetic element. Consistent with the selfish operon theory (110, 111), *mtrCAB* and its accessories exist as a succinct, contiguous cluster of genes, making it possible to transfer this metabolic capacity as a single functional package. Not only do the genes required for the reduction of specific electron acceptors often occur in close succession like *mtrCAB* (57, 112–115), the most phylogenetically distant transfers are typically limited to those encoding metabolic proteins (116, 117). In addition to enabling the easy mobilization of clusters like *mtrCAB*, this modular arrangement also minimizes disruption to other metabolic networks if suddenly lost from the genome (110, 111).

This still does not explain why *mtrCAB* appears to be missing from some genera or even entire phyla. The main limiting factor as to whether or not *mtrCAB* is maintained in a genome is not discernible from our present analyses, but studies of other mobile elements have revealed that their retention in a recipient genome is just as contingent on compatibility with the recipient’s ecology, physiology, and cell architecture as it is on phylogenetic proximity of the HGT donor species (118–120). This principle very likely explains why we did not detect *mtrCAB* in the genomes of Gram-positive bacteria, archaea, or eukaryotes; organisms cannot mature and assemble an outer membrane cytochrome complex like MtrCAB without an outer membrane to which it can be localized. Should one of these organisms that lack an outer membrane receive *mtrCAB* through HGT, the protein products would have to be adapted to fit into a very different kind of cell envelope, and the evolutionary time (or cost) required for these changes to arise may be too large for the genes to be retained in the new host’s genome, even if they would incur a fitness boost in the long term. That said, if these accommodations to the cell envelope have arisen in some organisms lacking an outer membrane, it is very likely that our current detection method—which is certainly biased toward MtrCAB as it exists in Gram-negative bacteria—would have missed these extremely diverged MtrCAB sequences.

**FIG 7:**
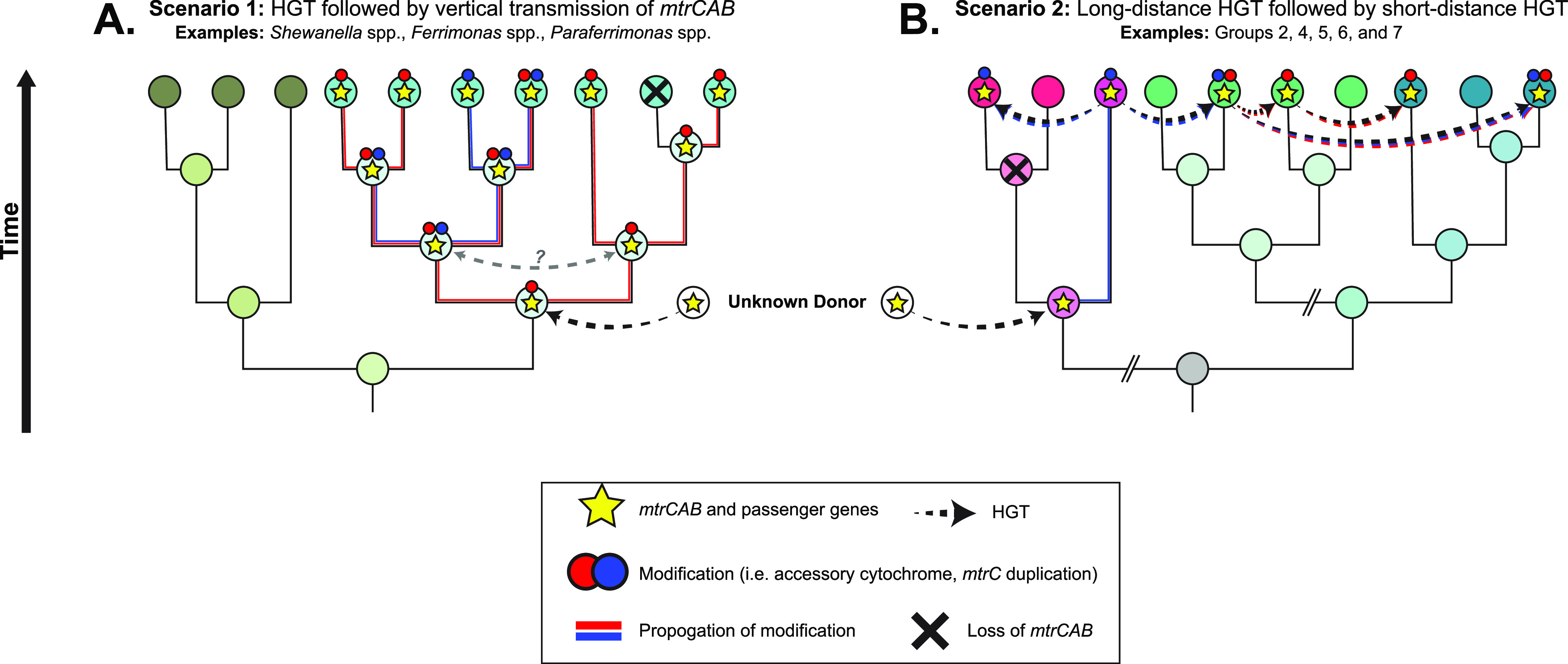
A hypothetical model representing two possible modes of *mtrCAB*’s dissemination to the species identified in our study.

Beyond the limits of cell envelope architecture and physiological capacity for cytochrome maturation, the factors determining the genomic retention of *mtrCAB* are not known. Among the *Shewanella* spp., in which *mtrCAB* was likely vertically disseminated (Fig. 7A), the species S. denitrificans and *S. violaceae* provide two independent examples of possible environment-dependent conditional dispensability of EET (121). *S. violaceae* was isolated from the upper layers of deep-sea sediment, and genomic analysis suggests that it has shifted from a CymA-dependent anaerobic metabolism to an aerobic one, facilitated by inhabiting the oxygenated sediment-water interface (82). S. denitrificans was similarly isolated from an oxic-anoxic interface in the central Baltic Sea and is capable of denitrification (83).

In regards to the retention of *mtrCAB* received by horizontal transfer, any adaptive advantage lent by MtrCAB could be voided by detrimental pleiotropic effects (120) or mismatch between *mtrCAB*’s regulatory regions and the new host’s transcriptional machinery could lead to deleterious overexpression of *mtrCAB* (119), or the selection pressure may be too weak to drive retention or is altogether limited by genetic drift before it can establish footing in a genome (122). Thus, when *mtrCAB* is received by horizontal gene transfer, any fitness cost incurred by the expression of these foreign genes would need to be minimal or resolvable through “domestication” of these genes and/or through compensatory evolution of other loci in the host genome that alleviates harm incurred by the foreign genes (120, 123), along with strong positive selection pressure for *mtrCAB* to remain in the genome (124).

Such adaptations are potentially represented in our findings as accessory cytochromes (Fig. 5; Table S1), MtrC diversifications (Fig. 6; Fig. S1 and S6), and the accompaniment of genetic modules involved in heme and cytochrome production (Fig. 3 and 4; Fig. S4 and S5). Once adapted to fit the ecophysiology of its new host, these changes would then be propagated to downstream HGT recipients and most likely be retained in recipients with an ecophysiology similar to that of its own. We speculate that this is what led to the relatively monofamilial MtrCAB clades like Groups 2, 4, 5, 6, and 7. The contemporary relationships observed in the MtrCAB tree (Fig. 2) may be the product of extensive HGT to various ancestral organisms and/or genetically and ecophysiologically similar species (Fig. 7B), as was similarly proposed by Zhong et al. (27). This may explain why some species in various genera lack *mtrCAB* while others have maintained or acquired the genetic potential for EET. However, as mentioned above in the case of *Shewanella* spp. and *Ferrimonadaceae*, it is still possible for *mtrCAB* to be vertically transmitted and then secondarily lost in some lineages (Fig. 7A).

### Revisiting the modularity of MtrCAB.

The data here provide insights into the diversification and thus potential adaptation of genes in the *mtr* gene cluster. Our analyses show that the core MtrAB module (Fig. S2 and S3) is relatively conserved, while the systems that support them (MtrC and accessory cytochromes) seem to vary (Fig. S1; Fig. 5 and 6). In contrast to the MtrC tree (Fig. S1), the individual protein trees for MtrA (Fig. S2) and MtrB (Fig. S3) rarely deviate from the topology and clade assignments in the MtrCAB tree (Fig. 2). This may be because the evolutionary trajectories of MtrA and MtrB are inextricably linked through molecular structure: any substantial change in one would break the entire MtrAB association without a parallel, compatible change in the other (28, 45). Conversely, because the majority of MtrC is relegated to the extracellular space and the only structural demands in relation to MtrAB are placement in the outer membrane and colocalization with MtrAB, MtrC may have more flexibility in its sequence and structure evolution. Indeed, this may be a corollary to the diverse functionality of MtrC in reducing a wide range of substrates, while MtrAB functions as an electron delivery system to MtrC.

This underscores the modularity of this system; MtrAB may be a core system that can be modified and adapted through the diversification and addition of MtrC homologs and other novel cytochromes. That said, our data on *mtrCAB*’s ubiquity, diversity, and the patterns that exist therein beg the question of how these various changes to the MtrAB ancillary system translate to function. Do these represent specialized components that operate optimally under different conditions? How do they affect the availability and nature of different extracellular electron acceptors and variations in their redox potential, crystallinity, and solubility? Accordingly, we can turn to the research on S. oneidensis MR-1 and other *Shewanella* spp., which have been studied in the lab for decades.

Previous studies differentiating MtrCAB, MtrDEF, and MtrC family proteins allow us to explore the relationship between modularity, evolution, and their associated function as they relate to Mtr-facilitated EET. OmcA, UndA, MtrG, and MtrH are not predicted to form a complex with MtrAB homologs (100, 101) but instead are thought to be reduced extracellularly by MtrC or MtrF anchored in their respective outer membrane conduits. The functional role of these ancillary extracellular cytochromes in metal reduction may be accessory, as *Shewanella* species mutants lacking only *omcA* or *undA* are still capable of EET (108) and these genes are transcribed from promoters separate from *mtrCAB* and *mtrDEF* (97). Genetic and biophysical analysis suggests that substrate specificity could be an accessory function for these proteins (31, 101). OmcA, for example, is thought to enhance adherence to solid substrates like electrodes (31, 125), hematite (105, 126), and goethite (127), while UndA may specialize in facilitating electron transfer to soluble substrates like ligand-bound Fe^3+^ (102, 108). MtrCAB and MtrDEF may be adapted to different conditions as well, as MtrCAB is preferentially expressed under iron-limited or O_2_-limited conditions, while MtrDEF prevails under iron-replete conditions or when cells are aggregated (128–130).

Revisiting our domain-wide data through the same lens of diversification and adaptation suggests that the modular deviations from the core *mtrCAB* model could similarly represent condition-specific adaptations. These questions and principles extend beyond MtrCAB to other systems utilizing MtrAB homologs at their core, such as DmsEF in extracellular dimethyl sulfoxide (DMSO) respiration (131), PioAB in phototrophic iron oxidation (35, 36), and MtoAB implicated in chemolithoautotrophic iron oxidation (40, 43, 132). The latter two instances may lead one to think that MtrC is responsible for conferring reductive capacity to an otherwise presumably oxidatively inclined MtrAB/MtoAB/PioAB core, especially because MtoA/PioA and MtoB/PioB do not form a separate “function-specific” clade on the individual MtrA and MtrB trees but instead group with MtrA and MtrB homologs belonging to complete MtrCAB modules (Fig. S2 and S3). Similarly, the distribution of proteins associated with iron-oxidizing and -reducing bacteria encoded genomically adjacent to *mtrCAB* (Fig. 5) and *mtoAB* (38, 43, 44) may hint at a possible evolutionary model for the functional diversification of Mtr as an oxidizing or reducing system. However, laboratory experiments have shown that MtrCAB of S. oneidensis MR-1 can be coerced to transmit electrons in the opposite direction of its traditional anodic ways (53, 54). In parallel, Bücking et al. showed that point mutations in MtrA and MtrB can rescue iron-reducing capability in an S. oneidensis mutant devoid of outer membrane cytochromes (133). Thus, even with our expanded catalog of MtrCAB and the changes that have accompanied it throughout its distribution to different lineages, we still lack sufficient functional data to inform the physiological capacity conveyed by these changes. We encourage future studies to focus on assessing whether electron flow directionality is a consequence of machinery, metabolism, environmental chemistry, or a combination of all.

A unifying quest in the field of geobiology is to understand the coevolution of life and Earth, and our findings further signify the importance of this pursuit. In light of the central role that known EET-capable organisms such as *Shewanella*, *Geobacter*, *Desulfuromonas*, and *Rhodopseudomonas* spp. play in key elemental cycles such as iron, sulfur, manganese, and carbon cycling (22, 134, 135), it is appropriate to consider whether among this vast diversity of taxa that the MtrCAB system plays a role comparable to that of the aforementioned microorganisms. At the moment, we do not know how the newly identified microorganisms use the Mtr system, nor do we know what role the environment has played in the diversification of MtrCAB and its associated machinery, nor under what conditions these adaptations have arisen. Many of the organisms identified in our study live at the oxic-anoxic interface, where they are faced with fluctuating oxygen concentrations and consequent changes in mineral solubility and redox potential. Mtr-linked EET may serve as an adaptation that permit r-selected strategists to persist in these types of habitats. Furthermore, the global supply of Fe(II), Fe(III), and O_2_ and the linked cycling of other elements have changed dramatically over the past 4 billion years, and prior to the rise of O_2_, Fe(II) was possibly one of the most important electron donors for anoxygenic photosynthesis (6, 7, 9, 136, 137). Should these adaptations in MtrCAB reflect changes in the environment like shifts in redox chemistry, understanding the timing of their emergence may shine light on major biogeochemical transitions in Earth’s history and address the first-order question of whether they can even be implicated in the ancient biogeochemical cycles that transformed Earth’s surface and habitability.

## MATERIALS AND METHODS

### Sequence retrieval.

MtrA (WP_011706573.1), MtrB (WP_011706574.1), and MtrC (WP_164927685.1) protein sequences from Aeromonas hydrophila were queried against the National Center for Biotechnology Information’s database of nonredundant protein sequences available on 28 July 2020 using PSI-BLAST.

### Data curation.

Individual amino acid sequences identified through PSI-BLAST were first filtered based on the presence or absence of non-Mtr domains, as determined by NCBI’s Conserved Domain Database search tool (138). Hits with additional detected protein domains were removed from subsequent alignments and tree building. The genomic loci for the remaining curated protein coding sequences were then compared to assess whether they comprised a genuine *mtrCAB* gene cluster. Of the total protein hits, any 2 coding sequences that were within 3,500 bp on a genome were marked as part of a single cluster. Those that did not meet this criterion were removed from further analysis. Clusters that did not have a complete set of the three proteins MtrCAB (in any order on the genome) were also removed. In the cases of metagenomes, MtrCAB clusters that comprised the majority of a contig or scaffold (i.e., 3 out of 11 or fewer total genes) were removed in the interest of maintaining a high confidence in the taxonomic assignments of each cluster. Putative cytochrome-encoding genes with synteny with the *mtrCAB* loci were identified with NCBI’s Conserved Domain Database search tool, and predicted cellular location was determined using PSORTb 3.0 (78).

Some species had more than one strain or sequenced genome represented at this stage of data curation, in which case one strain or genome was selected at random to remain for further analyses, while the others were removed. *Shewanella* and *Vibrio* hits without a species designation (e.g., *Shewanella* sp. or *Vibrio* sp.) were also discarded to avoid oversampling these relatively highly sequenced genera. The genomic order of the remaining *Shewanella* Mtr coding sequences in each cluster was examined; *Shewanella* clusters encoding MtrABC were labeled D-E-F, based on the delineation between MtrCAB and MtrDEF established in S. oneidensis MR-1 (25).

Additional MtrC/OmcA family proteins that were encoded next to a complete *mtrCAB* cluster were identified within strains encoding MtrCAB. S. oneidensis MtrC (WP_011071901.1), S. oneidensis MtrF (WP_011071903.1), S. oneidensis OmcA (WP_011071902.1), S. piezotolerans MtrH (WP_020913331.1), *S. loihic*a MtrG (WP_011866320.1), S. putrefaciens UndA (WP_011789901.1), *Niveibacterium* sp. COAC-50 MtrC (WP_172203423.1), *Gammaproteobacteria* bacterium sp. SP163 MtrC (MBA55444.1), and *Wenzhouxiangella* sp. XN201 MtrC (WP_164230597.1) were queried against a curated database of proteins from strains encoding MtrCAB. Any identified MtrC/OmcA coding sequences had to be located next to *mtrCAB* or to an additional *mtrC/omcA* family protein that was part of a larger *mtrCAB* gene cluster. Duplicates, hits with an E value of  >1 × 10^−10^, and proteins with additional detected protein domains were removed.

### MtrCAB maximum likelihood tree.

Alignments of the MtrA(D), MtrB(E), and MtrC(F) from each gene cluster were generated with ClustalΩ (139) and subsequently concatenated. Several different amino acid substitution models were originally tested—WAG, JTT, and LG—with or without gamma rate heterogeneity, invariant site testing, or both. The WAG amino acid substitution matrix (140) was chosen for subsequent tree-building based on its Bayesian information criterion (BIC) relative to other models tested. The maximum likelihood (ML) tree was built from a neighbor-joining (NJ) tree, followed by optimization and bootstrap support calculation in R using the phangorn package (141). The final tree was visualized with iTOL (142). Individual ML trees of MtrA, MtrB, and MtrC family proteins were also generated. Additional MtrC/OmcA family outer membrane decaheme- and undecaheme-encoding genes identified in the *mtr* gene clusters were included in the MtrC family ML tree but were not in the concatenated MtrCAB tree. MtoA and MtoB coding sequences (CDS) from the iron-oxidizing bacteria Gallionella capsiferriformans ES-2 and Sideroxydans lithotrophicus ES-1 (40, 143), as well as the PioA and PioB CDS from the photoferrotroph Rhodopseudomonas palustris TIE-1 (37), were included in the MtrA and MtrB ML trees, respectively.

### Whole-genome comparisons.

Genomes from the same genera encoding MtrCAB and genomes lacking MtrCAB were compared for genomic evidence indicative of potential horizontal gene transfer events. Any individual or combined instance of putative encoded transposases, syntenic tRNA-encoding genes, and genomic inversions and deletions were considered potential evidence for horizontal gene transfer. Genomes were downloaded from NCBI in June 2020 and then aligned using Progressive Mauve with automatically calculated seed weight and minimum LCB scores (144) using the Geneious Prime 2020.2 plug-in. Alignments were visualized for publication using EasyFig (145) and annotated in Adobe Illustrator 2020.
